# Genomics of Flower Identity in Grapevine (*Vitis vinifera* L.)

**DOI:** 10.3389/fpls.2019.00316

**Published:** 2019-03-21

**Authors:** Fabio Palumbo, Alessandro Vannozzi, Gabriele Magon, Margherita Lucchin, Gianni Barcaccia

**Affiliations:** Department of Agronomy, Food, Natural Resources, Animals, and Environment, University of Padua, Legnaro, Italy

**Keywords:** ABCDE genes, whorls, MADS, blooming, anthesis

## Abstract

The identity of the four characteristic whorls of typical eudicots, namely, sepals, petals, stamens, and carpels, is specified by the overlapping action of homeotic genes, whose single and combined contributions have been described in detail in the so-called ABCDE model. Continuous species-specific refinements and translations resulted in this model providing the basis for understanding the genetic and molecular mechanisms of flower development in model organisms, such as *Arabidopsis thaliana* and other main plant species. Although grapevine (*Vitis vinifera* L.) represents an extremely important cultivated fruit crop globally, studies related to the genetic determinism of flower development are still rare, probably because of the limited interest in sexual reproduction in a plant that is predominantly propagated asexually. Nonetheless, several studies have identified and functionally characterized some ABCDE orthologs in grapevine. The present study is intended to provide a comprehensive screenshot of the transcriptional behavior of 18 representative grapevine ABCDE genes encoding MADS-box transcription factors in a developmental kinetic process, from preanthesis to the postfertilization stage and in different flower organs, namely, the calyx, calyptra, anthers, filaments, ovary, and embryos. The transcript levels found were compared with the proposed model for *Arabidopsis* to evaluate their biological consistency. With a few exceptions, the results confirmed the expression pattern expected based on the *Arabidopsis* data.

## Introduction

Grapevine (*Vitis vinifera* L.) represents one of the most cultivated fruit crops on a global scale, with a production reaching approximately 75 million tons of berries and overlaying approximately 7.5 million hectares (OIV, 2016). Based on statistics from the OIV, wine is the main product of viticulture (68%), followed by fresh grapes for consumption (30%), raisins (1.8%), and minor products, such as juices, jellies, ethanol, vinegar, grape seed oil, tartaric acid, and fertilizers (0.2%). Considering the high economical value of global viticulture products, the lack of information and studies related to the genetic control of flower development and, more widely, to grapevine reproduction is quite surprising. In reality, the relatively limited interest of the scientific and producer communities on this issue is the main consequence of the static nature of viticulture, at least for what concerns the “old world.” The European and, on a larger scale, the global wine industry, is mainly focused on a few major cultivars, markedly restricting the range of genetic solutions for yield and quality improvement and relying almost exclusively on the improvement and optimization of management and oenological techniques. Moreover, although in recent years, novel varieties have been introduced to the market, as exemplified by the recent introduction of the first 10 disease-resistant grapevines produced in Italy by the University of Udine and the Institute of Applied Genomics (IGA) or by the PIWI (*pilzwiderstandsfähig* or fungus-resistant grape varieties) obtained by crossing European grape varieties and American or Asian fungus-resistant varietals, there is still a strong conservatism in viticulture, which is predominantly based on the use of clonally propagated traditional varieties.

Despite this, the complete understanding of the genetic mechanisms underlying the flowering process remains of primary importance since it profoundly affects winemaking and grapevine production. In fact, each stage of flower formation is critical for the development of the resulting population of berries. Examining the anatomy of the *Vitis* spp. reproductive system, wild *V. vinifera*, along with some American and Asian species, are dioecious with either male and female flowers, whereas all the main varieties employed for grape and wine production have inflorescences characterized by hermaphroditic flowers (Battilana et al., 2013). In this latter case, the conical panicle-shaped grapevine inflorescence is characterized by three levels of branching along the rachis, and triplets of flowers (dichasium) represent the last level of this complex structure. From the outside, each flower is characterized by four concentric whorls: sepals, petals, stamens, and carpels. The calyx represents the outermost ring and is formed by five sepals, while the calyptra or cap is a modified epidermal tissue composed of five joined petals. Although both structures play a protective role toward the inner reproductive whorls, the calyx is a permanent layer, while the cap is released when the pollen is mature. The androecium is organized in five stamens that, in turn, are each composed of a long filament ending with a bilocular anther. The anther, containing four pollen sacs, consists of three layers: the tapetum, endothecium, and epidermis. Finally, the gynoecium (or pistil) is the innermost layer. The stigma, responsible for pollen reception; the style, through which the pollen tube grows; and the ovary, where four ovules are protected and compartmentalized in two locules; represent the three major components of the pistil (Carmona et al., 2008; Vasconcelos et al., 2009).

The specification of such floral organs is controlled by a complex genetic regulatory network that acts in a coordinated way through a set of promotive and antagonistic iterations (Vasconcelos et al., 2009). This whole multitude of processes is synthesized and articulated into the “ABC model” (Coen and Meyerowitz, 1991), which links the overlapping expression patterns of homeotic genes to specific structures that are arranged in the four aforementioned whorls (Weigel and Meyerowitz, 1994). This model initially included only the homeotic genes of classes A, B, and C, but later it was extended to also include genes belonging to classes D and E (Jordan, 2006). The specific interactions that aid in the function of these genes lead to the differentiation of each specific flower whorl by encoding MADS-domain transcriptional factors, and in one case an AP2 TF (Irish, 2017). In more detail, the A-class genes specify the identity of the sepals (first whorl) when expressed alone and the petals (second whorl), when expressed in combination with the B-class genes (Jack, 2004). Moreover, they were found to repress the C-class genes in these whorls (Coito et al., 2018). The C-class genes alone specify the carpel identity, whereas in combination with some B-genes, they specify the stamen identity (Coen and Meyerowitz, 1991). It was demonstrated that the C-class genes repress the A-class genes in the third and fourth whorl; thus, A- and C-gene activities are mutually repressive (Jack, 2004). The D-class genes, together with some C-class genes, are involved in ovule identity specification within the carpel (Favaro et al., 2003; Carmona et al., 2008; Vasconcelos et al., 2009). The discovery of the importance of some genes later grouped in the E-class led to a revision of the ABC model (Honma and Goto, 2001; Theißen, 2001): these genes, discovered and characterized only recently due to genetic redundancy and overlapping functionality (Vasconcelos et al., 2009) are expressed in all floral whorls (Boss et al., 2002) and seem to function redundantly, forming complexes with A, B, C, and D proteins (Vandenbussche et al., 2003; Castillejo et al., 2005).

In the last 15 years, the molecular and genetic bases of floral development have been mainly investigated through the studies on three dicots: *Antirrhinum majus, Petunia hybrida*, and *Arabidopsis thaliana* (Jack, 2004). Studies on *Arabidopsis*, in particular, produced such a contribution to the research that, even today, its floral development model has been translated to a wide range of plant species with agro-economic importance (Coen and Meyerowitz, 1991; Fornara et al., 2003). Compared to the information available for the ABCDE model in *Arabidopsis*, data on the genetic and molecular processes involved in the grapevine reproductive phase remain limited (Carmona et al., 2008). Only a few genes have been functionally characterized, and based on their expression pattern, they were associated with certain stages of development and specific processes. Nonetheless, the grapevine ontogenetic mechanisms of organ formation and development are quite different with respect to other annual herbaceous or woody polycarpic plants (Carmona et al., 2007a,b), making it an extremely interesting system for the study of specific aspects of plant reproductive development.

Taking advantage of a recent reclassification of the grapevine MADS-box gene family performed by Grimplet et al. (2016), we provided a transversal approach to ABCDE model-involved genes by a transcriptional point of view focusing on the *V. vinifera* cv Pinot noir genotype, characterized by monoecious plants and hermaphroditic flowers. In particular, we evaluated the expression of 16 grapevine MADS-box orthologs in different floral tissues and at different time points before and after anthesis to ascertain whether the transcriptional behavior of these genes is in agreement with that observed in *Arabidopsis* and other plant species.

## Materials and Methods

### Plant Material and Sample Collection

Grapevine samples (*V. vinifera* L. cv Pinot noir, clone 115, grafted onto Kober 5BB rootstock) were collected from a germplasm collection Guyot-trained vineyard established in 2009 and located in the experimental farm “Lucio Toniolo” in Legnaro (PD) (45°21′5,68″N 11°57′2,71″E, -8 m above the sea level) during the growing season 2017/2018. The soil texture was as follows: 46% sand, 24% clay, and 30% loam; pH = 7.9; electric conductivity, 112 μS; and organic carbon, 1.1%. With the aim of following the main stages of flower development in different whorls both before and after anthesis, we considered seven time points over a time range of 22 days. Five samplings were performed before anthesis (50% of caps off), which took place on May 22nd, whereas two additional samplings were performed after anthesis. More precisely, preanthesis samples were collected 14 (I), 11 (II), 8 (III), 6 (IV), and 1 (V) days before flowering, whereas postanthesis samples were collected 6 (VI) and 8 (VII) days after flowering when fecundation had already occurred. At each time point considered, three inflorescences from three different plants were sampled and suddenly frozen in liquid nitrogen. For each inflorescence a consistent pool of single flowers was collected, each of which was dissected in the relative whorls with the aid of a scalpel and under a stereomicroscope. Flowers collected from preanthesis inflorescences were dissected into the calyx, ovary, anthers, anther filaments, and cap, whereas those collected after anthesis were dissected into the calyx, ovary, and embryos.

### RNA Extraction and cDNA Synthesis

For each sample, whorl-variable amounts of tissue were ground in liquid nitrogen, and total RNA was extracted using the Spectrum^TM^ Plant Total RNA Kit (Sigma-Aldrich, United States) following the manufacturer’s instructions. RNA quality and quantity were checked by means of conventional electrophoresis and spectrophotometry using a NanoDrop-1000 (Thermo Fisher Scientific). cDNA was synthetized starting from 500 ng of RNA using the Invitrogen^TM^ SuperScript^TM^ IV VILO^TM^ Master Mix (Thermo Fisher Scientific) according to the manufacturer’s instructions.

### RT-qPCR Expression Analyses

Sixteen grapevine ABCDE genes were selected as described by Grimplet et al. (2016) based on their orthology with *A. thaliana* genes and confirmed by means of a BLASTN approach. Primers were designed using the Primer-BLAST program of the National Center of Biotechnology Information (Rockville Pike, Bethesda, MD, United States) with melting temperatures between 58.83°C and 62.01°C, a length between 19 and 23 bp and finally with an amplicon length between 71 and 180 bp. The PN40024 accessions selected, together with the specific oligonucleotide sequences are reported in Supplementary Table S1. All the RT-qPCRs were performed on a StepOnePlus Real Time PCR system following the PowerUp SYBR Green Master Mix method (Applied Biosystems, Foster City, CA, United States). Each reaction was carried out in a volume of 10 μL, which contained 5 μL of SYBR Green, 1.2 μL of forward primer, 1.2 μL of reverse primer, 0.6 μL of sterilized water, and 2 μL of 1:10-diluted cDNA as a template. The run method set was as follows: initial denaturation 95°C for 20 s, followed by 40 cycles of denaturation at 95°C for 3 s and primer annealing, extension and gathering the fluorescence signal at 60°C for 30 s. Subsequently, the melting curve analysis was achieved to verify the specificity of the primer with the following program: 95°C/15 s, 60°C/1 min, and 95°C/15 s. The baseline and threshold cycles (Ct) were automatically determined by the software of the system. Three technical replicates were taken in each biological replicate. The ubiquitin-conjugated enzyme gene (VIT_08s0040g00040) was used as an internal control. The relative expression level for all selected genes was calculated using the QGene method (Muller et al., 2002).

## Results and Discussion

### Class-Specific Expression Levels

**FIGURE 1:**
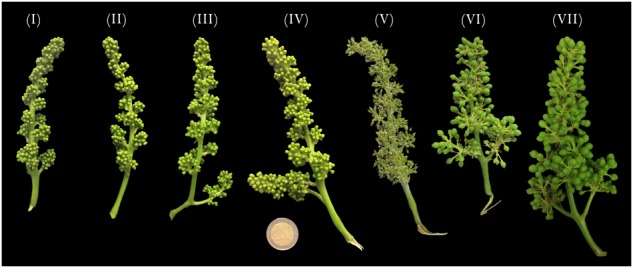
*Vitis vinifera* cv Pinot noir inflorescences sampled at 14 (stage I), 11 (stage II), 8 (stage III), 6 (stage VI), and 1 (stage V) days prior to anthesis and at 6 (stage VI) and 8 (stage VII) days after anthesis. Samples were collected in three propagations of the same clone.

In 2016, Grimplet et al. (2016) performed a true genome-wide analysis of the whole set of MADS-box genes in grapevine based on the v1 and v2 predictions (CRIBI, University of Padua, Padua, Italy) of the 12X PN40024 grapevine reference genome (Jaillon et al., 2007). All the 90 MADS-box genes were named according to the Super-Nomenclature Committee for Grape Genome Annotation (sNCGGa) (Grimplet et al., 2014) and include all those TFs belonging to the ABCDE model. We utilized this new classification to select 16 genes known for their involvement in flower organ identity in *Arabidopsis* and, for some of them, in grapevine (Boss et al., 2003; Carmona et al., 2007b) and screened their behavior in seven stages of flower development over a range of different tissues/organs. Inflorescences from three different plants of *V. vinifera* cv Pinot noir were collected in correspondence to the late G-stage (Baggiolini scale, Baggiolini and Keller, 1954) at 14 (I), 11 (II), 8 (III), 6 (IV), and 1 (V) days before flowering, whereas postanthesis samples were collected 6 (VI) and 8 (VII) days after flowering when fecundation had already occurred (J-stage, Baggiolini scale) (Figure 1). Subsequently, flowers were dissected into the calyx, ovary, anthers, anther filaments, and cap in preanthesis (stages I–V), and the calyx, ovary, and embryos in postanthesis (stages VI and VII) and screened for the expression of selected ABCDE genes. The nomenclature of the selected genes was based on those of Grimplet et al. (2016), together with the orthologs in *Arabidopsis*; the PN40024 12x v1 IDs and some additional information is reported in Table 1.

**Table 1:** List of homeotic genes considered in the present study.

Gene class	*Vitis* gene^∗^	Previous name	PN40024 v1 ID	Best *Arabidopsis* match
*Class A*	*VviAP1*	*VAP1* (Calonje et al., 2004)	VIT_01s0011g00100	AT1G69120, *AP1*/*AGL7*
	*VviFUL1*	–	VIT_17s0000g04990	AT5G60910, *FUL*/*AGL8*
	*VviFUL2*	*VFUL-L* (Calonje et al., 2004)	VIT_14s0083g01030	AT1G69120, *AP1*/*AGL7*
*Class B*	*VviAP3a*	*VvAP3* (Poupin et al., 2007)	VIT_18s0001g13460	AT3G54340, *AP3*/*ATAP3*
	*VviAP3b*	*VvTM6* (Poupin et al., 2007)	VIT_04s0023g02820	AT3G54340, *AP3*/*ATAP3*
	*VviPI*	*VvMADS9* (Sreekantan et al., 2006) *VvPI* (Poupin et al., 2007)	VIT_18s0001g01760	AT5G20240, *PI*
*B-sister*	*VviABS1*	–	VIT_10s0042g00820	AT5G23260.4, *TT16*/*ABS*/*AGL32*
	*VviABS2*	–	VIT_01s0011g01560	AT5G23260.3, *TT16*/*ABS*/*AGL32*
	*VviABS3*	–	VIT_02s0025g02350	AT5G23260.2, *TT16*/*ABS*/*AGL32*
*Class C*	*VviAG1*	*VVMADS1* (Boss et al., 2001)	VIT_12s0142g00360	AT3G58780, *SHP1*/*AGL1*
	*VviAG2*	–	VIT_10s0003g02070	AT4G18960, *AG*
	*VviAGL6a*	*VvMADS3* (Boss et al., 2002)	VIT_15s0048g01270	AT2G45650, *AGL6*
	*VviAGL6b*	–	VIT_16s0022g02330	AT2G45650, *AGL6*
*Class D*	*VviAG3*	*VvMADS5* (Boss et al., 2002)	VIT_18s0041g01880	AT4G09960, *STK*/*AGL11*
*Class E*	*VviSEP1*	*VvMADS2* (Boss et al., 2002)	VIT_14s0083g01050	AT5G15800, *SEP1*/*AGL2*
	*VviSEP2*	–	VIT_17s0000g05000	AT3G02310, *SEP2*/*AGL4*
	*VviSEP3*	VviMADS4 (Boss et al., 2002)	VIT_01s0010g03900	AT1G24260, *SEP3*/*AGL9*
	*VviSEP4*	–	VIT_01s0011g00110	AT3G02310, *SEP2*/*AGL4*

*For each gene, the corresponding nomenclature based on Grimplet et al. (2016) and previous literature, together with the PN40024 12X v1 ID and the relative *Arabidopsis* orthologs are indicated. The asterisk refers to gene nomenclature based on Grimplet et al. (2016).*

#### A-Class Genes

**FIGURE 2:**
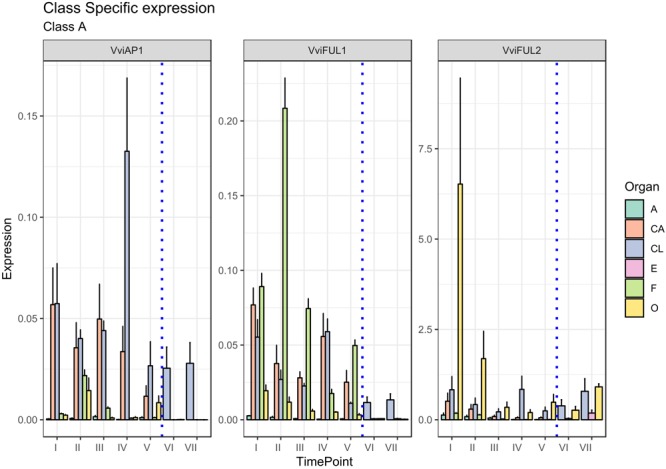
Expression level of A-class genes *VviAP1, VviFUL1*, and *VviFUL2*. Preanthesis samples were collected 14 (stage I), 11 (stage II), 8 (stage III), 6 (stage IV), and 1 (stage V) days before flowering, whereas postanthesis samples were collected 6 (stage VI) and 8 (stage VII) days after flowering when fecundation had already occurred. The blue dotted line indicates full anthesis. A, anther; CA, cap; CL, calyx; E, embryo; F, filament; O, ovary. Transcript levels were normalized to the expression of *UBIQUITIN* gene and plotted as normalized transcript expression. Bars indicate the SE of three biological replicates each one composed of three technical replicates.

The first class of genes considered in the present study is represented by Class-A, which encompass four major TFs, namely, *APETALA1* (*AP1*), *FRUITFULL1 (FUL1), FRUITFULL-like*, and an additional class-A gene designated AP2 (Theißen et al., 2016). The latter was not considered in the present study since it does not encode a MADS-box TF. *APETALA1* (*AP1*) has two main functions: the first is to specify the identity of sepals (first whorl), when expressed alone, and petals (second whorl), when expressed in combination with the B-class genes *APETALA3* (*AP3*) and *PISTILLATA* (PI; Jack, 2004); the second function is to repress the C-class genes in these whorls (Coito et al., 2018). The putative ortholog of *Arabidopsis AP1* was isolated in grapevine (*VAP1*) (Calonje et al., 2004) and corresponds to the gene *VviAP1* (VIT_01s0011g00100) based on a recent MADS-box classification (Grimplet et al., 2016). Based on previous results, its expression pattern seemed to differ substantially from that of *AP1* in *Arabidopsis*. In fact, *VAP1* was found to be expressed predominantly in stamens and developing carpels and to be excluded from the sepal-forming region soon after the meristem determination, not consistent with a function in sepal identity specification (Calonje et al., 2004; Carmona et al., 2008). These observations, together with other evidence obtained in other plant species, such as *A. majus* (Huijser et al., 1992) and *G. hybrida* (Yu et al., 1999), led to questions about the role of these genes in the specification of sepal identity and provided arguments to revise the involvement of *AP1* in class-A gene action (Litt and Irish, 2003). In reality, our results confirmed a high level of expression of *VviAP1* in both the calyx and cap compared to all other tissues analyzed (Figure 2), perfectly in agreement with previous observations in other plant species, including *Arabidopsis* (Bowman et al., 1993), *Camellia japonica* (Sun et al., 2014) and *Medicago truncatula* (Roque et al., 2018). Concerning the calyx, a high level of transcripts was detected at all time points considered, including the postanthesis stages, with the highest accumulation detected at 6 days prior to anthesis. A significant transcript level was also found in the cap (CA) organ, whose identity is specified by the joint action of *VviAP1* and B-class genes. In stamens (anthers and filaments) and in the carpel, *VviAP1* was almost absent and therefore consistent with mutually repressive activity of the A- and C-class genes (Jack, 2004). Our results are partially in contraposition with that observed by Coito et al. (2018), who evaluated the spatiotemporal expression of the same gene in male female and perfect grapevine flowers. Surprisingly, in that study, *VviAP1* expression was barely detected in the sepal and petal regions and restricted to the inner part of the flower. We believe the incongruence between these observations and the role of A-class genes was because the phenological stages considered were too precocious (from the B to G stage based on the Baggiolini scale) with respect to those considered in the present study (starting from the late G stage to J stage).

Another MADS-box gene belonging to the A-class is *FRUITFULL* (*FUL*), which was shown to play a role in carpel and fruit development contributing to the normal development of the gynoecium valve (Gu et al., 1998) and floral meristem identity redundantly with *AP1* (Ferrándiz et al., 2000). Based on the grapevine MADS-Box classification proposed by Grimplet et al. (2016), two *AtFUL* orthologs were selected: *VviFUL1* (VIT_17s0000g04990) and *VviFUL2* (VIT_14s0083g01030). The expression level of *VviFUL2* was consistent with that in the previous literature. In fact, the ovary was the organ showing the highest accumulation of this transcript, especially at 14 and 11 days prior to anthesis (stages I and II). *VviFUL2* corresponded to *VFUL*-L identified by Calonje et al. (2004), who observed, in agreement with our results, that the expression of *VviAP1* (*VAP1* in Calonje et al., 2004) and *VviFUL2* (*VFUL*-L in Calonje et al., 2004) was only partially overlapping. In fact, very early in flower development, the expression of *VFUL-L* becomes restricted to the carpel-forming region at the central part of the flower meristem and continues to be expressed at high levels through the early stages of fruit development (Calonje et al., 2004). More recently, the expression of *FUL*-like genes in carpels was also described in *Aquilegia coerulea* (Pabõn-Mora et al., 2013) and *C. japonica* (Sun et al., 2014).

More intriguing is the expression pattern of the other grapevine paralogues, namely, *VviFUL1*, whose expression was generally lower in terms of normalized transcript level if compared to *VviFUL2* but also showed significant expression in the filament and, to a lower extent, in the ovary at all stages prior to blooming. An interesting observation is the fact that the expression of this gene has significant overlap with the expression of *VAP1* described by Calonje et al. (2004).

#### B-Class Genes

B-class genes are delegates to specify petals and stamen identity through the joint expression with A- and C-class genes, respectively (Coen and Meyerowitz, 1991). In *Arabidopsis*, the B-class group is constituted by *APETALA3* (*AP3*) and *PISTILLATA* (*PI*), and their expression is in agreement with their role in the ontogenesis of the two aforementioned whorls. In grapevine, three orthologs of B-function genes have been characterized and correspond to *VviPI* (VIT_18s0001g01760), *VviAP3a* (VIT_18s0001g13460), and *VviAP3b* (VIT_04s0023g02820) according to Grimplet et al. (2016). In reality, based on phylogenetic analyses (Poupin et al., 2007; Coito et al., 2018), these genes cluster in three different clades: *PI, AP3*, and *TM6*, respectively. While *VviPI*, corresponding to *VvMADS9* and/or *VvPI* (Sreekantan et al., 2006; Poupin et al., 2007), forms a clearly separate clade, namely, the *PI* clade (Poupin et al., 2007; Coito et al., 2018), *VviAP3a* and *VviAP3b*, previously designated *VvAP3* and *VvTM6*, respectively (Poupin et al., 2007; Table 1), present differences at the level of specific C-terminal motifs (Vandenbussche et al., 2003).

**FIGURE 3:**
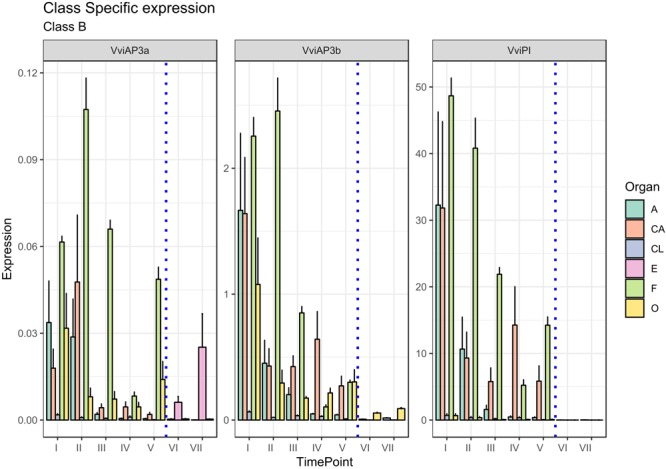
Expression level of B-class genes *VviAP3a, VviAP3b/VviTM6*, and *VviPI*. Preanthesis samples were collected 14 (stage I), 11 (stage II), 8 (stage III), 6 (stage IV), and 1 (stage V) days before flowering, whereas postanthesis samples were collected 6 (stage VI) and 8 (stage VII) days after flowering when fecundation had already occurred. The blue dotted line indicates full anthesis. A, anther; CA, cap; CL, calyx; E, embryo; F, filament; O, ovary. Transcript levels were normalized to the expression of *UBIQUITIN* gene and plotted as normalized transcript expression. Bars indicate the SE of three biological replicates each one composed of three technical replicates.

The role of these genes in petal and stamen identity was confirmed in our analysis (Figure 3). The highest *VviPI, VviAP3a*, and *VviAP3b* transcript levels were detected in the cap and stamen (anther and filament) tissues. Regarding *VviAP3a*, previous studies in a hermaphroditic variety of *V. vinifera* (Poupin et al., 2007), showed that the expression of this gene is restricted to petals and stamens, whereas *in situ* hybridization experiments indicated that the *VviAP3a* transcripts also localize in the carpels but are limited to early stages of flower development (Coito et al., 2018). In reality, a discrete expression of VviAP3a was also detected in carpels and embryos in our analysis. Similar results were observed in *Paeonia lactiflora*, where the B-class genes *PLAP3*-1 and *PLAP3*-2 were found to be strongly expressed in petals, stamen, and carpels (Gong et al., 2017).

A possible explanation for the increase in *VviAP3a* expression in embryos (stages VI and VII corresponding to 6 and 8 days postanthesis) (Figure 3) could be ascribed to the close relation intervening between B- and B-sister class genes, which are hypothesized to be redundantly involved in ovule identity specification and proven to reach high transcript levels in embryo integuments (Theißen and Melzer, 2006).

*TM6* is considered to be a B-class homeotic gene, and although an *Arabidopsis* ortholog was not identified, it was detected in other plant species, including *Solanum lycopersicum* flowers (de Martino et al., 2006) and *P. hybrida* (Rijpkema et al., 2006). It is worth noting that *VviAP3b in situ* hybridization detected this transcript not only in petals and stamens but also in the ovary. This observation is in agreement with our results indicating a discrete transcript accumulation on carpels in the first stage considered (I).

Concerning *PISTILLATA*, in *Arabidopsis*, this gene is expressed in cells that will give rise to petals, stamens and carpel primordia in the early stages of flower development (Goto and Meyerowitz, 1994; Sundström et al., 2006). Coito et al. (2018) observed an accumulation of the *VviPI* transcript in *Vitis* flower development in the center of the flower meristem, at early stages of flower development, but when sepals start to emerge, the highest expression was observed in cells that will develop into petals and stamens, remaining confined to the second and third whorls during the later stages of flower development. Our results, which considered even later stages of flower development, confirmed the high accumulation in the cap and stamens, but, interestingly, the highest level of transcript accumulation was detected in the filament tissue with respect to the anthers. Overall, as far as flower development proceeded and anthesis drew nearer, the expression of *VviPI* declined in all tissues considered.

#### B-Sister Genes

**FIGURE 4:**
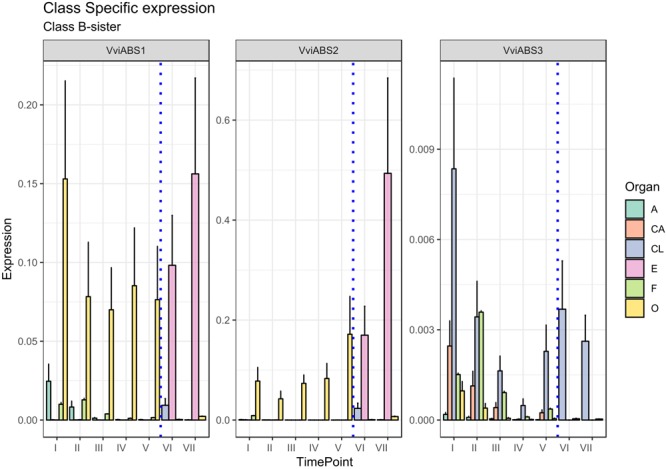
Expression level of B-sister class genes *VviABS1, VviABS2*, and *VviABS3*. Preanthesis samples were collected 14 (stage I), 11 (stage II), 8 (stage III), 6 (stage IV), and 1 (stage V) days before flowering, whereas postanthesis samples were collected 6 (stage VI) and 8 (stage VII) days after flowering when fecundation had already occurred. The blue dotted line indicates full anthesis. A, anther; CA, cap; CL, calyx; E, embryo; F, filament; O, ovary. Transcript levels were normalized to the expression of *UBIQUITIN* gene and plotted as normalized transcript expression. Bars indicate the SE of three biological replicates each one composed of three technical replicates.

There is another homeotic gene category closely related to the B-class genes: the B-sister class. MADS-box genes belonging to this cluster are phylogenetically close to the B-class genes and have been identified in all angiosperms and gymnosperms investigated so far showing highly conserved ovular expression (Becker et al., 2002; Chen et al., 2012). Genes belonging to this class, in contrast to those of the B-class, are expressed exclusively in female flower structures, in particular, in the integument tissues surrounding the ovules (Theißen and Melzer, 2006). *Arabidopsis B sister* (*ABS*) belongs to this gene class and, beyond having a proven function in seed pigmentation (Nesi et al., 2002), is hypothesized to have a role in ovule formation, acting in a complex and redundant interaction network. In rapeseed canola (*Brassica napus*), the *ABS* ortholog (*BnTT161-4*) was found to be involved in embryo and seed development, whereas in rice (*Oryza sativa*), *OsMADS29* is mainly expressed in the ovule and regulates the expression of pivotal genes involved in programmed cell death in the nucellar region of developing seeds (Yin and Xue, 2012; Chen et al., 2013). An exhaustive study on the transcriptional behavior of B-sister genes in grapevine still remains to be elucidated. Based on the PN40024 12X genome prediction, three MADS-box genes are phylogenetically related to *AtABS* in grapevine: *VviABS1* (VIT_10s0042g00820), *VviABS2* (VIT_01s0011g01560), and *VviABS3* (VIT_02s0025g02350) (Grimplet et al., 2016). Considering that ovaries sampled from stages I to V also included ovules, with the exception of *VviABS3*, which did not show a clear expression pattern in all tissues analyzed, *VviABS1* and *VviABS2* expression perfectly matched what was expected (Figure 4). In fact, the ovary in preanthesis (all stages) and embryo, were the only tissues where transcripts accumulated (Figure 4). It is likely that the high level of *VviABS1* and *VviABS2* transcripts in preanthesis ovaries is fully attributable to the presence of the ovules within the carpels.

#### C-Class Genes

**FIGURE 5:**
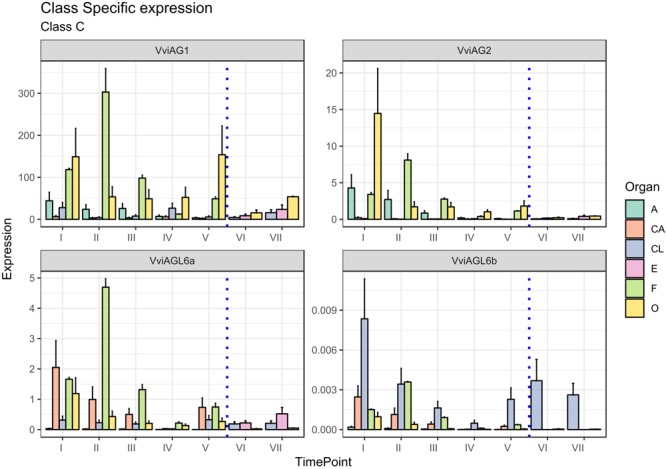
Expression level of C-class genes *VviAG1, VviAG2, VviAGL6a*, and *VviAGL6b*. Preanthesis samples were collected 14 (stage I), 11 (stage II), 8 (stage III), 6 (stage IV), and 1 (stage V) days before flowering, whereas postanthesis samples were collected 6 (stage VI) and 8 (stage VII) days after flowering when fecundation had already occurred. The blue dotted line indicates full anthesis. A, anther; CA, cap; CL, calyx; E, embryo; F, filament; O, ovary. Transcript levels were normalized to the expression of *UBIQUITIN* gene and plotted as normalized transcript expression. Bars indicate the SE of three biological replicates each one composed of three technical replicates.

The C-class gene *AGAMOUS* (*AG*) specifies carpel identity in model species, whereas in combination with *AP3* and *PI*, the gene specifies stamen identity (Coen and Meyerowitz, 1991). Two putative orthologs of the *AG* gene subfamily were identified in grapevine (Grimplet et al., 2016) and were designated *VviAG1* (VIT_12s0142g00360) and *VviAG2* (VIT_10s003g02070). Whereas *VviAG1* (VIT_12s0142g00360), which was previously cloned by Boss et al. (2001) now appears to be strictly related to *Arabidopsis SHP1*, a D-class gene (Theißen et al., 2016; Coito et al., 2018), *VviAG2* has not been considered in previous studies and seems to be the best candidate ortholog of the *Arabidopsis AGAMOUS* gene (AT4G18960) based on a recent reannotation of the TAIR database. Thus, we believe this study is the first one to consider the expression of grapevine *AGAMOUS* orthologs during flower development. Although having different functions in the determination of floral identity, the C- and D-class genes form a monophyletic MADS-box clade, known as the AG subfamily of MADS-box genes. Whereas phylogenetic analyses did not clearly assign *VviAG1* and *VviAG2* as class-C or class-D genes, expression analyses did, clearly showing a high level of transcript accumulation in stamens (anthers and filaments) and ovaries for both of them, suggesting their role as class-C factors (Figure 5). For this reason, we decided to consider *VviAG1* and *VviAG2* together.

A more detailed examination at the stage of *VviAG2* transcript accumulation showed that a marked peak was observed in the ovary 14 days prior to anthesis (stage I), and its transcription tended to decrease in this organ as anthesis got closer. A relatively high level of expression was also detected in anthers and filaments in the earlier stages considered here (stages I and II, corresponding to 14 and 11 days to anthesis). *VviAG1* showed a much higher transcript level compared to its paralogues, peaking in the ovary (stages I and V) and in the filament at stage II. Moreover, a moderate transcript level was maintained in the anthers overall during the whole developmental kinetic process. *VviAG1* showed an increasing expression in both the ovary and embryo during postanthesis, which is biologically consistent with a putative role in berry development (Carmona et al., 2008). It is worth noting that the *VviAG1* sequence corresponding to *VvMADS1* was identified in 2001 by Boss et al. (2001), who reported the expression of this gene in the two inner whorls, as well as during berry development. An interesting aspect emerging from our results is that *VviAG1* is moderately expressed in sepals (stages I and IV). This observation is not in agreement with the role of C-class genes. Nevertheless, similar results had already been observed in *P. lactiflora* where the PLAG ortholog of *VviAG1* was expressed in sepals (Gong et al., 2017). Moreover, Boss et al. (2002) showed that the overexpression of this gene in grapevine is associated with an altered sepal morphology raising the question of whether this gene could have a role in determination of this whorl or in the interaction with A-class genes.

The last two class-C genes considered were *VviAGL6a* (VIT_15s0048g01270) and *VViAGL6b* (VIT_16s0022g02330). Both *VviAGL6a* and *VviAGL6b* show homology to *Arabidopsis AGL6* and *AGL13*, whose function in flower development still remains to be elucidated (Boss et al., 2003; Ohmori et al., 2009; Schauer et al., 2009), although it was proven that AGL6 and AGL13 bind to AG, and for this reason, it can be involved in determining AG functional specificity (Fan et al., 1997; Hsu et al., 2014). Moreover, yeast two-hybrid studies in several plant species revealed that AGL6 and AGL13 proteins can interact with AP1/FUL-like, B-class, D-class, and SEP-like MADS-box proteins (Hsu et al., 2003; de Folter et al., 2005). Recently, it was suggested by Hsu et al. (2014) that AGL6 could act in a regulatory feedback as a repressor of *AGL13* involved in male and female gametophyte morphogenesis.

It’s worth pointing out that Hsu et al. (2014) recently described *AGL13* as a possible ancestor of the E-class genes. Considering the close phylogenetic relationship between AGL6 and AGL13, we cannot rule out the possibility that these genes belong to the E-class rather than the C-class.

*VviAGL6a* corresponds to the *VvMADS3* gene (Boss et al., 2002), whose expression was first detected in late inflorescence development with greater transcript levels in petals compared to the inner two whorls present. In agreement with this observation, *VviAGL6a* was expressed in the cap at the first stages considered, with a decrease until anthesis. It’s worth noting that, together with petals, the filament also showed a high accumulation of this transcript, reaching a peak at 11 days prior to anthesis (stage II). The expression of this gene in the filament has not been described before, but it must be considered that this is the first study examining the specific expression of this gene in this tissue. However, Rijpkema et al. (2009) already described the accumulation of the *VviAGL6a* ortholog *PhAGL6* in Petunia anthers. *VviAGL6b*, whose expression was never evaluated in a kinetic study of flower development, exhibited a pattern of expression, which is comparable to the one observed for *VviSEP2* (see “E-Class Genes” section), especially concerning the calyx. In this organ, the expression level of *VviAGL6b* decreased approaching anthesis and then increased again after fertilization. This is in agreement with the redundant and overlapping functionality of E-class genes.

#### D-Class Genes

**FIGURE 6:**
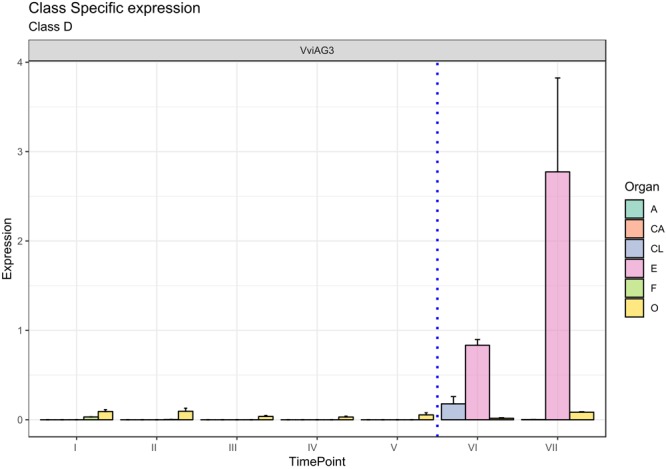
Expression level of D-class gene *VviAG3*. Preanthesis samples were collected 14 (stage I), 11 (stage II), 8 (stage III), 6 (stage IV), and 1 (stage V) days before flowering, whereas postanthesis samples were collected 6 (stage VI) and 8 (stage VII) days after flowering when fecundation had already occurred. The blue dotted line indicates full anthesis. A, anther; CA, cap; CL, calyx; E, embryo; F, filament; O, ovary. Transcript levels were normalized to the expression of *UBIQUITIN* gene and plotted as normalized transcript expression. Bars indicate the SE of three biological replicates each one composed of three technical replicates.

The D-class gene *SEEDSTICK* (*STK*), together with *AG*, is involved in ovule identity specification within the carpel (Favaro et al., 2003; Carmona et al., 2008; Vasconcelos et al., 2009). The closest accession in grapevine was designated *VviAG3* (VIT_18s0041g01880). Figure 6 clearly shows that *VviAG3* is totally switched-off in all whorls considered except for the ovary, where it is poorly expressed. Conversely, *VviAG3* appeared to be strongly induced with an increase in the embryo, in agreement with that reported by Boss et al. (2002), who observed a strong expression of this gene (namely, *VvMADS5*) in mature carpels, developing seeds and pre- and postveraison berries. Recently, a target resequencing of this gene in a collection of 124 grapevine cultivars showed that a point variation causing the arginine-197-to-leucine substitution was fully linked to stenospermocarpy (Royo et al., 2018).

#### E-Class Genes

The involvement of E-class genes in flower identity was discovered relatively recently as a consequence of their high genetic redundancy and overlapping functionality (Vasconcelos et al., 2009). The discovery of the importance of the *SEPALLATA* (*SEP*) genes, namely, *SEP1, SEP2, SEP3*, and *SEP4*, led to a revision of the first ABC model (Honma and Goto, 2001; Theißen, 2001). The *SEP* genes play a crucial role in petal, stamen, and carpel formation. In fact, all the flower whorls of the *sep1*/*sep2*/*sep3* triple mutant develop into sepals and flowers that become indeterminate (Pelaz et al., 2000). Vegetative leaves, rather than sepals, are formed in the *sep1*/*sep2*/*sep3*/*sep4* quadruple mutants (Ditta et al., 2004). In grapevine, based on the new classification of Grimplet et al. (2016) four gene predictions were associated with the E-class group, namely, VIT_14s0083g01050, designated *VviSEP1*, VIT_17s0000g05000, designated *VviSEP2*, VIT_01s0010g03900 designated *VviSEP3*, and finally VIT_01s0011g00110, corresponding to *VviSEP4*.

**FIGURE 7:**
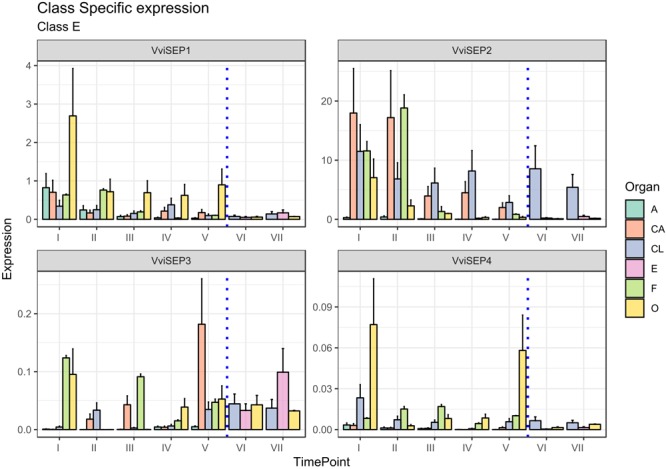
Expression level of E-class genes *VviSEP1, VviSEP2, VviSEP3*, and *VviSEP4*. Preanthesis samples were collected 14 (stage I), 11 (stage II), 8 (stage III), 6 (stage IV), and 1 (stage V) days before flowering, whereas postanthesis samples were collected 6 (stage VI) and 8 (stage VII) days after flowering when fecundation had already occurred. The blue dotted line indicates full anthesis. A, anther; CA, cap; CL, calyx; E, embryo; F, filament; O, ovary. Transcript levels were normalized to the expression of *UBIQUITIN* gene and plotted as normalized transcript expression. Bars indicate the SE of three biological replicates each one composed of three technical replicates.

The ortholog of *Arabidopsis SEP1*, namely, *VviSEP1* (VIT_14s0083g01050), designated *MADS2* based on Boss et al. (2002) was previously found to be expressed early during flower development until anthesis in all the whorls except for sepals. As shown in Figure 7, the overall expression of the *VviSEP1* gene is detectable in all the floral whorls, at least for the first stages considered (stages I and II), an observation consistent with the facts that the *Arabidopsis* orthologs *SEP1*-*4* have been postulated to specify all whorl identities by complexing A-, B-, C-, and D-class proteins (Vandenbussche et al., 2003; Castillejo et al., 2005) and that its expression is totally shut down after fecundation occurred (stages VI and VII). The ovary is the organ showing the highest expression of *VviSEP1* in all preanthesis stages (from I to V) in agreement with that observed by Boss et al. (2002) examining the expression of *VvMADS2*.

Similar to *VviSEP1, VviSEP2* showed a generalized expression in all organs in the first stages except for the anthers (stages I–III), with a decrease in all tissues except for the calyx, whose expression lasted over fertilization. The persistence of *VviSEP2* expression in the calyx over the whole kinetic process described here is surprising, considering these genes are mainly required for the activity of the B- and C-class floral homeotic genes, and the triple *sep1*/*2*/*3* mutants produce sepals in the place of all floral organs (Castillejo et al., 2005).

*SEP3* is involved in sepal, petal, stamen, carpel, and ovule development, and its ectopic expression is sufficient to activate *AtAP3* and *AtAG* (Pelaz et al., 2000; Favaro et al., 2003; Ditta et al., 2004). In *Vitis*, the expression of *VviSEP3* prior to anthesis was slightly confusing, with transcript accumulation detected in filaments and the ovary and calyx depending on the stage. An interesting aspect is the increasing transcript accumulation observed in petals from stages I to V (except for stage IV). This expression pattern could suggest a possible role of this gene in the development and maturation of these organs. Something similar was hypothesized for anther *SEP* gene, namely, *FaMADS9* in strawberry (Seymour et al., 2011). Of special interest is the postanthesis expression detected in ovary and embryo tissues, which is in agreement with previous observation performed by Boss et al. on this gene [*VvMADS4* in Boss et al. (2002)]. These observations are not unexpected considering that *SEP* orthologs were found to be pivotal for the normal ripening of berries, such as strawberry (Seymour et al., 2011) and in tomato (*S. lycopersicum*; Ampomah-Dwamena et al., 2002).

Finally, *VviSEP4*, whose function was not previously investigated, showed its highest expression in the ovary at stages I and V, although a basal and constant accumulation was also detected in the calyx and filaments. As a general observation, it must be noted that the expression of *VviSEP3* and *VviSEP4* appeared to be 1–2 orders of magnitude lower compared to those of *VviSEP1* and *VviSEP2*. In *Arabidopsis*, the closest orthologous gene, *AtSEP2*, was functionally demonstrated to determine the identity of carpels and stamens (Pelaz et al., 2000).

### The Grapevine ABCDE Model

**FIGURE 8:**
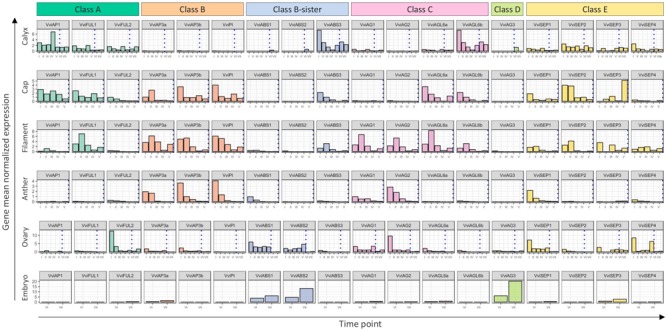
General overview of the expression of the 18 MADS-box genes in different tissues analyzed. Transcript levels were standardized on the mean expression of each gene in all tissues and stages considered. Preanthesis samples were collected 14 (stage I), 11 (stage II), 8 (stage III), 6 (stage IV), and 1 (stage V) days before flowering, whereas postanthesis samples were collected 6 (stage VI) and 8 (stage VII) days after flowering, when fecundation already occurred. The blue dotted line indicates full anthesis.

The “ABCDE” model maintains that class A+E genes specify sepals, A+B+E specify petals, B+C+E specify stamens, C+E specify carpels, and C+D+E specify ovules. According to the recent nomenclature of MADS-box TFs performed by Grimplet et al. (2016), we selected 18 genes belonging to these different classes and determined their expression in different organs and time points. Figure 8 illustrates the mean standardized expression value of all the MADS-box genes in the six different whorls considered in the present study. The standardization on the mean expression of each single gene among all the time points and tissues allowed better comprehension of the contribution of different classes to the development of a particular organ. Based on our findings, a complex of three class-A genes, namely, *VviFUL1, VviFUL2*, and *VviAP1* together with the four class-E genes, determines calyx identity in grapevine flower (Figure 8). It is worth noting that two other genes, namely, *VviAGL6b* and *VviABS3*, belonging to class-C and the class B-sister, respectively, were significantly expressed in sepals throughout the whole kinetic process considered. Based on the *Arabidopsis* model, these genes should not be involved in the identity of the sepals. Nevertheless, regarding *VviAGL6b*, it must be considered that several authors questioned its membership in class-E rather than its function as a class-C MADS box (Hsu et al., 2014).

The three class-A genes, designated as *VviFUL1* and *VviAP1*, and, to a lesser extent *VviFUL2*, were coexpressed with the B-class genes *VviAP3a, VviAP3b*, and *VviPI* in petals. Together with these genes, a significant transcript accumulation was also detected for the class-E genes *VviSEP1-3* and the C-class genes *VviAGL6a* and *VviAGL6b*. In addition, in this case, the expression pattern of the latter genes suggests their putative role as class-E genes rather than as class-C ones (Figure 8).

Concerning the third whorl, a complex of three class-B genes, i.e., *VviAP3a, VviAP3b*, and *VviPI*, plus the two class-C genes, *VviAG1* and *VviAG2*, were expressed in both filaments and anthers, whereas *VviAGL6a* and *VviAGL6b* were detected only in filaments (Figure 8). Concerning the class-E genes, all 4 *VviSEP* genes showed a discrete expression in the filament, while only *VviSEP1* was detected in anthers. It is worth mentioning the ectopic expression of the class-A gene *VviFUL1* and class-B-sister gene *VviABS3* in the filament, which has never been reported in the previous literature (Figure 8).

In the inner whorl, the carpel, together with the expected expression of class-C (*VviAG1* and *VviAG2*) and class-E genes (*VviSEP1, VviSEP3*, and *VviSEP4*), a high level of expression for class-A gene *VviFUL2* and class-B-sister genes (*VviABS1* and *VviABS2*) was detected. The involvement of the latter two class-genes in the ovary is probably because, during the first five stages (I–V), ovules were not separated from ovaries; thus, the expression of ovule-specific genes in ovary is a consequence of a copresence of both organs in the samples used for transcriptional analyses.

Finally, looking at gene expression in embryos, we found the genes belonging to the class-B-sisters *VviABS1* and *VviABS2* and the class-D gene *VviAG3*, as expected based on the ABCDE model.

**FIGURE 9:**
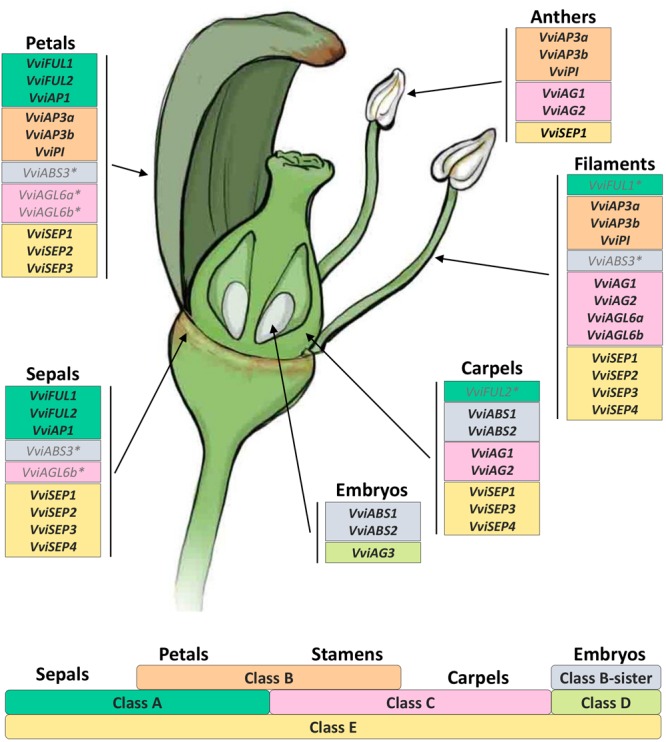
The floral model and the underlying ABCDE model of organ identity determination in *V. vinifera* L. The bottom part of the figure shows the genetic ABCDE model. According to this model, organ identity during flower development in the model organism *A. thaliana* is controlled by five classes of floral homeotic genes providing overlapping floral homeotic functions: A, B, C, D, and E. A+E genes specify sepals, A+B+E specify petals, B+C+E specify stamens, C+E specify carpels, and C+D+E specify ovules. In the upper part of the figure we reported all genes belonging to different classes according to their expression in different flower organs analyzed. Asterisks indicate genes whose expression was not expected in a given whorl based on the floral ABCDE model.

Expression data obtained over a developmental kinetic process and represented in Figure 8 enabled us to develop a grapevine model adapting our transcriptional evidence to the ABCDE model of organ identity determination in *A. thaliana*. Figure 9 shows our proposed ABCDE model for grapevine. What is remarkable, although with several slight differences, is its robustness. In fact, it seems that the overall logic of the process is also conserved in grapevine. Identifying all the MADS-box genes involved in flower identity and analyzing their behavior in different whorls and developmental stages represents a first step in the understanding of grapevine flower genetics and biology. We believe that the present study could provide a starting point for a better comprehension of many aspects directly or indirectly related to flower and berry biology. Among these aspects are the determinants of large seasonal variation in grape yield, the sex determination in monoecious and dioecious species, phenomena related to seedset such as apireny and stenospermocarpy but also to fruit set, such as millerandage or the gradient in berry maturation within the cluster. Some of these aspects, such as those related to berry maturation and quality, have been investigated more thoroughly, given their economic importance for the wine industry and table grape production, while some have been just recently been taken into account, such as the flower sex determination (Coito et al., 2018) and stenospermocarpy (Royo et al., 2018). Some others have been fairly ignored.

## Conclusion

The aim of this work was to translate the ABCDE model verifying the coherence between *Arabidopsis* and grapevine in terms of flower identity gene expression. The results revealed that the majority of genes investigated follow the expression pattern expected based on the model, being detected, alone or in combination, in the floral whorls where they were supposed to be accumulated. Some of the genes considered here had already been functionally characterized, but many merit investigation to ascertain their functional conservation with their *Arabidopsis* MADS-box orthologs. In particular, it will be very interesting to focus on the B-sister genes and E-class genes, whose expression and functionality merit further investigation. We believe that, starting from these robust transcriptional evidences, new functional genomic tools available nowadays will greatly contribute to deeply understand the contribution of single and combined TFs in the determination of flower identity in grapevine.

## Author Contributions

GB and ML designed the research. GM and FP conducted and controlled the experiments and analyzed the data. AV carried out the bioinformatics analyses. FP and AV wrote the manuscript. All authors contributed to editing the manuscript.

## Conflict of Interest Statement

The authors declare that the research was conducted in the absence of any commercial or financial relationships that could be construed as a potential conflict of interest.
